# Preceding Wound Complications as Drivers of Surgical Site Infection after Bypass for Chronic Limb-Threatening Ischemia

**DOI:** 10.3400/avd.oa.25-00156

**Published:** 2026-08-19

**Authors:** Masaya Sano, Takuya Hashimoto, Rina Matsumoto, Takashi Endo, Satoshi Yamamoto, Juno Deguchi

**Affiliations:** Department of Vascular Surgery, Saitama Medical Center, Saitama Medical University, Kawagoe, Saitama, Japan

**Keywords:** chronic limb-threatening ischemia, surgical site infection, wound complications

## Abstract

**Objectives:**

Surgical site infection (SSI) remains a serious complication following bypass surgery for chronic limb-threatening ischemia (CLTI). This study aimed to characterize wound complications (WCs) preceding SSI and to identify predictors of SSI in a Japanese CLTI cohort with a high prevalence of dialysis.

**Methods:**

A single-center retrospective study was conducted in patients undergoing infra-inguinal bypass with saphenous vein grafts (2012–2021). WCs and subsequent SSI within 3 months were evaluated. Predictors of SSI were examined using the Mann–Whitney U test for continuous variables and the χ^2^ tests for categorical variables.

**Results:**

Among 148 patients, the incidence of SSI was 8.7% (n = 13). Preceding WCs included lymphorrhea/lymphocele (n = 7), hematoma (n = 4), and edema (n = 2). Hematoma had the highest conversion rate to SSI (25.0%). *Staphylococcus aureus* was the predominant pathogen. Univariate analysis identified female sex as a potential predictor of SSI (*p* = 0.03), while dialysis dependence (43.9% of the cohort) was not associated with SSI risk.

**Conclusions:**

SSI following bypass surgery in CLTI predominantly arises from preceding WCs, characterized by edema or inadequate drainage of blood or lymph. Targeted management to prevent these preceding WCs may be a crucial strategy to reduce SSI.

## Introduction

Surgical site infection (SSI) following bypass surgery for chronic limb-threatening ischemia (CLTI) remains a serious postoperative complication with implications for graft loss, amputation risk, and mortality.^1–5)^ Unlike contaminated or organ-related surgery, lower limb bypass is classified as class I clean surgery; therefore, SSIs commonly arise in pre-existing wound complications (WCs) rather than from deep contamination.

Several characteristics make CLTI bypass wounds vulnerable: (1) extensive lymphatic disruption associated with saphenous vein harvest and target artery exposure; (2) impaired tissue perfusion that could lead to necrosis and delayed healing despite revascularization; (3) antithrombotic therapy and heparin use, which increase bleeding propensity; and (4) postoperative edema promoting cellulitis or delayed healing.^6,7)^ Based on these characteristics, SSI following bypass surgery in CLTI is not an independent event, but rather an infection resulting from preceding WCs. While numerous studies have identified risk factors for SSI after lower limb bypass, including female sex, older age, obesity, renal failure, diabetes, anticoagulation therapy, prolonged operative time, blood loss, and intraoperative transfusion,^5,8–12)^ in Japan, CLTI patients exhibit an exceedingly high rate of dialysis dependence, which differs from patient backgrounds reported elsewhere. This study aimed to characterize WCs preceding SSI and to identify predictors of SSI in a Japanese CLTI cohort with a high prevalence of dialysis dependence.

## Materials and Methods

### Study design

This retrospective observational study was approved by the Saitama Medical Center Ethics Committee (no. 2022-088). Consecutive patients undergoing infra-inguinal bypass using saphenous vein grafts between 2012 and 2021 were included. Patients with a chronic course of acute limb ischemia or vasculitis were excluded from the analysis, as were those who did not have wound follow-up for at least 30 days postoperatively. Predictors of SSI were examined based on previously reported risk factors.

### Perioperative and wound management

Vein harvest was performed using a skin-bridge technique. All wounds were closed in two or more layers with absorbable sutures, and the skin was closed with interrupted nylon sutures and staples. A padded film dressing was applied for 48 hours. Prophylactic antibiotics (cefazolin or ampicillin–sulbactam) were administered for up to 48 hours. If a different antibiotic (e.g., vancomycin or piperacillin–tazobactam) had been used empirically or as a treatment based on culture results before surgery, the same antibiotic was normally continued throughout the perioperative period. Antibiotics to treat foot infections were continued after surgery as needed. All patients received overnight heparin after surgery, and oral antithrombotic medication was resumed or initiated the following day. Wounds were evaluated daily during hospitalization and photographed weekly. Computed tomography was performed one week after surgery to assess subcutaneous fluid collection. Drainage was undertaken only when infection was evident. After discharge, patients were followed as needed every one to four weeks for up to three months, during which wounds were examined and photographed at each visit. If an SSI occurred, the abscess or fluid was drained, and necrotic or infected tissues were removed. Wound cultures were performed when patients underwent interventions. Antibiotics were started empirically or as broad spectrum therapy depending on the condition of the wounds and the patient. After identification of cultures, antibiotics were switched accordingly. Standard dressings were used for wounds. Negative pressure wound therapy (NPWT) was not performed.

### Definitions

This analysis focused on WCs that could precede SSI, specifically lymphorrhea, lymphocele, hematoma, and edema. Lymphorrhea was defined as lymphatic leakage persisting for more than 48 hours after surgery, and lymphocele was defined as a subcutaneous lymphatic fluid collection. Hematoma referred to clinically apparent blood collection. Edema was evaluated as a potential substrate for cellulitis or delayed healing and was defined as clinically apparent swelling of the operated limb that developed after revascularization and persisted for several months postoperatively, as documented in the medical records. We confirmed that this swelling was not attributable to deep venous thrombosis, active infection, or systemic causes such as cardiac, renal, or hepatic insufficiency. Dehiscence caused by SSI or intentional drainage was not included. SSI was defined as an infection developing in a WC that required intervention such as debridement, drainage, or antibiotic therapy.

### Statistical analysis

All analyses were performed using JMP Pro 16. Due to the small size of the cohort, continuous variables were expressed as median values and compared using the Mann–Whitney U test. On the other hand, categorical variables were compared using χ^2^ tests. A *p*-value <0.05 was considered statistically significant.

## Results

### Patient characteristics

Of the 187 patients who underwent infra-inguinal bypass during the study period, 10 were diagnosed with acute limb ischemia or vasculitis, 29 lacked adequate postoperative wound follow-up (4 died, 25 were transferred), and were excluded, leaving 148 patients for analysis. The median age was 75 (IQR: 68–80) years, and 22.9% were female. Diabetes mellitus was present in 72.2% of patients, hemodialysis dependence in 43.9%, and 72.2% had a history of smoking. Preoperative antithrombotic therapy was dual antiplatelet therapy in 8.7% and anticoagulant therapy in 19.5%. Baseline characteristics of patients are shown in **Table 1**.

**Table 1:** Patient baseline characteristics

Variables	n = 148
Age (years), median (IQR)	75 (68–80)
Female, n (%)	34 (22.9%)
Body mass index (kg/m^2^), median (IQR)	22.0 (19.9–24.3)
Hypertension, n (%)	113 (76.3%)
Diabetes Mellitus, n (%)	107 (72.2%)
Dyslipidemia, n (%)	63 (42.5%)
Hemodialysis, n (%)	65 (43.9%)
Ischemic heart disease, n (%)	56 (37.8%)
Cerebrovascular disease, n (%)	43 (29.0%)
Smoking history, n (%)	107 (72.2%)
Statin, n (%)	35 (23.6%)
SAPT, n (%)	125 (84.4%)
DAPT, n (%)	13 (8.7%)
Anticoagulation therapy, n (%)	29 (19.5%)
Rutherford classification, n (%)	
4	12 (8.1%)
5	108 (73.0%)
6	28 (18.9%)
WIfI stage, n (%)	
2	16 (10.8%)
3	33 (22.3%)
4	99 (66.9%)

IQR, interquartile range; SAPT, single antiplatelet therapy; DAPT, dual antiplatelet therapy

The median operative time was 379 (IQR: 321–439) min, and median blood loss was 99 (IQR; 50–210) ml. Antibiotics were administered preoperatively in 24.3% of cases, and continued postoperatively in 20.2%. The rate of antibiotic use increased with increasing WIfI stage.

The great saphenous vein was used as the conduit in 95.3% of cases, including 22 in situ. Proximal anastomoses were located in the groin region in 68.9% of patients, and 86.4% of distal anastomoses were below the knee. One patient died within 3 months after surgery from pneumonia. Five patients experienced early graft occlusion within 3 months. Two required major amputation and 3 underwent re-intervention, including a jump bypass, thrombectomy, or graft repair (**Table 2**).

**Table 2:** Surgical data and 3-month outcomes

Surgical data	
Operative time (minutes), median (IQR)	379 (321–439)
Blood loss (ml), median (IQR)	99 (50–210)
Intraoperative transfusion, n (%)	5 (3.3%)
Preoperative antibiotic use, n (%)	36 (24.3%)
WIfI stage 2, n/total	1/36
WIfI stage 3, n/total	3/36
WIfI stage 4, n/total	32/36
Postoperative antibiotic use, n (%)	30 (20.2%)
WIfI stage 2, n/total	0/30
WIfI stage 3, n/total	2/30
WIfI stage 4, n/total	28/30
Inflow revascularization, n (%)	32(21.6%)
FF bypass, n/total	3/32
Iliac EVT, n/total	10/32
TEA, n/total	19/32
Proximal anastomosis	
CFA, proximal SFA, PFA (groin incision)	102 (68.9%)
Mid SFA-AKPopA (thigh incision)	8 (5.4%)
BKPopA	31 (21.0%)
Other	7 (4.7%)
Distal anastomosis	
AKPopA	2 (1.4%)
BKPopA	18 (12.2%)
Distal to PopA	128 (86.4%)
Graft type	
GSV harvest	119 (80.4%)
GSV in situ	22 (14.9%)
SSV harvest	7 (4.7%)
3-month outcomes	
SSI	13 (8.7%)
Death	1 (0.6%)
Occlusion	5 (3.4%)
Amputation	2 (1.4%)
Re-intervention	3 (2.0%)

IQR, interquartile range; FF bypass, femorofemoral crossover bypass; EVT, endovascular therapy; TEA, thromboendarterectomy; CFA, common femoral artery; SFA, superficial femoral artery; PFA, profunda femoral artery; AKPopA, above-knee popliteal artery; BKPopA, below-knee popliteal artery; GSV, great saphenous vein; SSV, small saphenous vein; SSI, surgical site infection

### Summary of SSI cases

SSI occurred in 13 patients, corresponding to an incidence of 8.7% (**Table 2**). The median (IQR) time from surgery to SSI onset was 19 (14–29) days. All SSI cases in this study developed at the graft harvest site rather than at the anastomotic site. The distribution of SSI sites demonstrated that infections developed in the groin in 15.4% of cases, in the thigh in 23.1%, and in the lower leg in 61.5% of patients. *Staphylococcus aureus* was the most common bacterium detected in wound cultures of SSI patients, followed by coagulase-negative *Staphylococcus* (CNS). Both are Gram-positive cocci, which are part of the normal skin flora. There were no SSI-related deaths or amputations during the 3 months follow-up. However, graft rupture occurred in one case (**Supplementary Table 1**; case No. 9) due to infection spreading to the graft, requiring repair with sartorius muscle coverage (**Table 3**).

**Table 3:** Summary of surgical site infection cases

SSI	
Time to onset, median days (IQR)	19 (14 –29)
Distribution of SSI site	
Groin	2 (15.4%)
Thigh	3 (23.1%)
Lower leg	8 (61.5%)
Bacteria detected from wound cultures	
MSSA	5 (38.4%)
MRSA	3 (23.0%)
Coagulase negative *Staphylococcus*	2 (15.3%)
Enterococcus	1 (7.7%)
Klebsiella	1 (7.7%)
No growth	1 (7.7%)
No data	3 (23.0%)
3-month outcomes (SSI-related)	
Amputation	0 (0%)
Death	0 (0%)
Graft rupture	1 (7.7%)

SSI, surgical site infection; IQR, interquartile range; MSSA, methicillin-sensitive *Staphylococcus aureus*; MRSA, methicillin-resistant *Staphylococcus aureus*

### Preceding WCs

WCs preceding subsequent SSI included lymphorrhea or lymphocele in 7 cases, hematoma in 4, and severe edema in 2. Hematoma showed the highest likelihood of progression to SSI, with a conversion rate of 25.0%. Lymphatic complications also demonstrated a notable progression to infection, with a conversion rate of 14.9%. Edge necrosis and noninfected wound dehiscence did not lead to SSI in any patient (**Table 4**).

**Table 4:** Incidence of wound complications and conversion rate for surgical site infections

	Incidence	SSI conversion (case/total)
Lymphorrhea/Lymphocele, n (%)	47 (31.7%)	14.9% (7/47)
Hematoma, n (%)	16 (10.8%)	25.0% (4/16)
Edema, n (%)	148 (100%)	1.3% (2/148)
Dehiscence, n (%)	29 (19.5%)	0% (0/29)
Edge necrosis, n (%)	6 (4.0%)	0% (0/6)

SSI, surgical site infection

### Exploration of SSI predictors

**Table 5** presents an analysis of SSI risk factors using univariate analysis. Although female sex (*p* = 0.03) was associated with SSI, other reported risk factors were not. Due to insufficient sample size, multivariate analysis could not be performed in this study.

**Table 5:** Analysis of factors associated with surgical site infection

Factor	SSI, n = 13	No SSI, n = 135	P value
Age (years), median (IQR)	71 (61 –80)	75 (69–80)	0.21
Female, n (%)	6 (46.1%)	28 (20.7%)	0.03
Body mass index (kg/m^2^), median (IQR)	23.5 (21.9–24.2)	21.8 (19.7–22.4)	0.15
Diabetes Mellitus, n (%)	11 (84.6%)	96 (71.1%)	0.30
Hemodialysis, n (%)	7 (53.8%)	59 (43.7%)	0.86
Smoking history, n (%)	7 (53.8%)	100 (74.1%)	0.27
DAPT, n (%)	1 (7.6%)	12 (8.8%)	0.88
Anticoagulation therapy, n (%)	2 (15.3%)	27 (20.0%)	0.68
Operative time (minutes), median (IQR)	394 (320–476)	377 (321–434)	0.57
Blood loss (ml), median (IQR)	130 (65–240)	95 (50–210)	0.55
Intraoperative transfusion, n (%)	1 (7.6%)	4 (2.9%)	0.42
Preoperative antibiotic use, n (%)	2 (15.3%)	34 (25.1%)	0.43
Postoperative antibiotic use, n (%)	2 (15.3%)	28 (20.7%)	0.64

IQR, interquartile range; DAPT, dual antiplatelet therapy; SSI, surgical site infection

## Discussion

This study demonstrated that SSIs following infra-inguinal bypass for CLTI occurred predominantly in pre-existing WCs, particularly hematoma and lymphatic leakage. Although univariate analysis identified female gender as a significant predictor of SSI, the small sample size renders this finding exploratory. Nevertheless, the novelty of this study lies in the identification of a consistent process based on meticulous daily wound observation: all observed SSIs had preceding WCs. This provides granular clinicopathological insights that are often overlooked in large-scale registry studies.

There have been various reports investigating SSIs and WCs after bypass surgery for CLTI, but most of them discussed SSI and WCs as phenomena of the same rank. However, because of the unique characteristics of wounds following bypass surgery, an SSI occurs when an infection has spread in the preceding WCs, as defined in this study. We found that poor drainage of lymphatic fluid or coagula at the wound site, especially lymphocele and hematoma, were the most common causes potentiating SSI. We hypothesize that normal skin bacteria invade the fluid-retaining area immediately below the wound, leading to infection. This hypothesis is supported by our finding that *S. aureus*, a common skin bacterium, was the most frequently detected organism in wound cultures in this study. Furthermore, continuous antibiotics use increased as the WIfI stage increased from preoperative to postoperative. However, continuous antibiotics use after surgery did not suppress SSI occurrence. This finding also supports our hypothesis that infection occurs when bacteria invade fluid retention areas where antibiotics may have limited penetration.

Previous reports from Western countries, where the prevalence of dialysis is relatively low, have indicated that the incidence of SSI among CLTI patients ranges from 4% to 20%, with dialysis identified as a significant predictor of SSI.^12)^ Conversely, in Japan, a country with a notably high prevalence of dialysis, a previous study involving a cohort with 40% dialysis patients reported a 15% SSI incidence but found that dialysis was not a predictor of SSI.^5)^ Similarly, in the present study involving a cohort with 43.9% dialysis patients, the SSI incidence was 8.7%, and dialysis did not emerge as a predictor of SSI in univariate analysis. The discrepancy between Western and Japanese findings regarding dialysis as a risk factor may be attributed to the high background prevalence of dialysis, which statistically diminishes its relative impact as a predictor.

Hematoma showed the most frequent progression to infection, likely due to retained blood providing a favorable milieu for bacterial growth. Though a previous study reported that anticoagulation therapy was a risk factor of SSI,^9)^ in this study, anticoagulation therapy was associated with hematoma (*p* = 0.01; **Supplementary Table 2**), but not with SSI (*p* = 0.68) in our univariate analysis. Lymphorrhea and lymphocele, similar to hematoma, predispose patients to infection through persistent fluid accumulation and impaired wound healing. Although hyperemia, increased microvascular permeability, reperfusion-associated inflammation, and lymphatic disruptions are considered potential causes of postoperative edema in previous reports,^13)^ its precise pathophysiology remains poorly understood. In this study, postoperative edema was inevitable after revascularization, yet only two cases progressed to cellulitis and subsequent SSI. While edema and impaired wound healing contributed to dehiscence, no case resulted in SSI, likely due to the absence of fluid accumulation beneath the wound. Although extensive necrosis throughout the wound was not found in this study, necrosis only at the wound edges was observed in a few cases, but it did not result in SSI.

Previous research has generally concluded that male sex poses a higher risk of SSI.^14)^ However, SSI occurrence was substantially higher in females following heart and vascular surgery.^15)^ In previous vascular surgery studies,^4,9)^ authors have argued that differences in fat distribution may be a contributing factor, as women typically have more fat tissue in their hips and thighs.^16)^ Other possible explanations include comorbidities, local skin flora, or hormonal influences, but the underlying reasons remain unclear.^15)^ Although this study identified female sex as a potential SSI predictor, the small sample size precluded the analysis to a univariate approach, rendering the findings exploratory.

To date, the discussions of SSI after bypass surgery have grouped cases together without a detailed examination of their underlying pathology. However, the consequences of SSI can range from minor to severe outcomes, such as graft loss and amputation. Kobayashi et al. reported that graft rupture can occur due to infection spreading from SSI, resulting in poor outcomes.^5)^ In our case, graft rupture also arose in a similar manner. Although the clinical impact was limited in that case, it is important to recognize that the prognosis is highly dependent on the underlying pathology process. Additionally, regarding the distribution of SSI in this study, the incidence was highest in the lower leg. This likely reflects the fact that 87% of distal anastomoses in this cohort were performed below the knee, resulting in a higher density of incisions in the lower leg. Moreover, the lower leg may experience more severe edema and higher skin tension than the thigh, potentially compromising wound healing. Nevertheless, additional studies are needed to substantiate these findings. The present study investigated how WCs can act as a trigger for SSI. Although WCs (lymphorrhea/lymphocele, hematoma, and edema) are associated with surgical manipulation and postoperative changes, making it challenging to address them, the results of this study suggest that preventing preceding WCs could reduce the frequency of SSI. The effectiveness of NPWT in preventing SSI has been demonstrated in various settings,^17–19)^ but there are few reports on whole bypass wounds (not only the groin) for CLTI. While further evidence is needed, prophylactic NPWT could be considered an exploratory option to mitigate preceding WCs by effectively managing dead space and subcutaneous fluid.

### Limitations

Findings of this study should be interpreted in light of several limitations. The sample size was small, which may have limited the statistical power. Additionally, twenty-nine patients were excluded due to insufficient wound follow-up. Because transferred patients (n = 25) often have more complex clinical needs, their exclusion may have led to a systematic underestimation of the actual SSI and WC incidence. Another limitation is that the SSI definition in this study differed from the CDC guideline by focusing on clinical need for intervention. Consequently, the SSI rate may have been underestimated in this study. Subclinical lymphoceles not detectable by clinical examination or CT may have been missed. Combining lymphorrhea and lymphocele could obscure the true conversion rates. Edema was assessed based on subjective clinical findings rather than objective circumferential measurements, which may have introduced ascertainment bias. Finally, the number of SSIs was small, limiting the study’s power to assess associations with clinical outcomes.

## Conclusion

In this study, WCs preceding SSI after bypass surgery were attributed to edema or insufficient drainage of blood or lymph fluid. Mitigating these preceding WCs may serve as a strategy to reduce SSI incidence, although further studies are required to confirm this relationship.

## Supplementary Materials

Supplementary Table 1 Summary of 13 SSI cases.

Supplementary Table 2 Hematoma and antithrombotic therapy.
