# Supplementary material for: Preceding Wound Complications as Drivers of Surgical Site Infection after Bypass for Chronic Limb-Threatening Ischemia
Source: Ann Vasc Dis. 2026 Aug 19;19(1):25-00156. doi: 10.3400/avd.oa.25-00156 (PMC13494578; doi:10.3400/avd.oa.25-00156)
Supplement: Supplementary file 1 [file avd-19-1-25-00156-s001.pdf]

Supplementary Table 1 Summary of 13 SSI cases

No	Gender	SSI site	Preceding WCs	Culture	Onset(day)
1	F	Lowe Leg	Hematoma	MSSA	11
2	M	Thigh	Lymphorrhea/Lymphocele	MRSA	19
3	F	Lowe Leg	Hematoma	CNS	15
4	F	Groin	Lymphorrhea/Lymphocele	MSSA	13
5	M	Groin	Lymphorrhea/Lymphocele	MRSA	14
6	M	Lowe Leg	Lymphorrhea/Lymphocele	ND	23
7	F	Lowe Leg	Hematoma	No growth	26
8	F	Lowe Leg	Edema	MSSA	32
9	M	Thigh	Lymphorrhea/Lymphocele	MSSA	17
10	F	Lowe Leg	Edema	ND	19
11	M	Thigh	Hematoma	MRSA, CNS	11
12	M	Lowe Leg	Lymphorrhea/Lymphocele	*Enterococcus*	32
13	M	Lowe Leg	Lymphorrhea/Lymphocele	ND	14

SSI, surgical site infection; Onset, onset from surgery; F, female; M, male; MSSA, methicillin-sensitive *Staphylococcus aureus*; MRSA, methicillin-resistant *Staphylococcus aureus*; CNS, coagulase negative *Staphylococcus*; ND, no data

Supplementary Table 2 Hematoma and antithrombotic therapy

	Hematoma, n=16	No Hematoma, n=132	P value
SAPT, n (%)	12 (75%)	113 (85%)	0.29
DAPT, n (%)	3 (18%)	10 (8%)	0.18
Anticoagulation therapy, n (%)	7 (43%)	22 (16%)	0.01

SAPT, single antiplatelet therapy; DAPT, dual antiplatelet therapy
